# Individual mangrove trees provide alternative reef fish habitat on backreefs

**DOI:** 10.1038/s41598-024-69524-y

**Published:** 2024-08-10

**Authors:** Hannah von Hammerstein, Theresa-Marie Fett, Sebastian C. A. Ferse, Véronique Helfer, Stuart Kininmonth, Sonia Bejarano

**Affiliations:** 1 Faculty of Biology and Chemistry (FB2), University of Bremen, Bibliothekstraße 1, 28359 Bremen, Germany; 2 Leibniz Centre for Tropical Marine Research (ZMT), Fahrenheitstraße 6, 28359 Bremen, Germany; 3 Department of Geography & Environment, University of Hawai’i at Mānoa, Honolulu, HI 96822 USA; 4 School of Marine Studies, The University of the South Pacific, Suva, Fiji; 5 Heron Island Research Station, The University of Queensland, Brisbane, Australia; 6 Present Address: Faculty of Fisheries and Marine Sciences, Bogor Agricultural University (IPB), Jl. Agatis 1, Dramaga Campus, Bogor, 16680 Indonesia; 7 Marine Ecology Department, Faculty of Biology and Chemistry, University of Bremen, Leobener Strasse, 28359 Bremen, Germany

**Keywords:** Ecosystem ecology, Tropical ecology, Ecology, Environmental sciences

## Abstract

Mangrove trees occur in a variety of geomorphic and sedimentary settings. Yet, studies investigating their role as habitat providers often focus on the most common biophysical types, such as deltaic, estuarine, open coast or lagoonal mangroves on soft sediments, disregarding less typical environments. Here, we investigated the influence of individual mangrove trees growing on a consolidated backreef system (Laucala Bay, Fiji) on habitat use by reef fishes. Combining field surveys and an experiment, we quantified the extent to which reef mangrove trees serve as habitat for solitary or shoaling reef fishes. Using mangrove tree mimics, we disentangled effects attributable to the physical structure of trees from those related to their bio-chemical properties. We found that fish numbers were 3.7 times higher within close proximity to the mangrove trees than at control sites and correlated significantly with root system perimeter. The roots of larger trees sheltered aggregations of juveniles and adults at incoming and high tides. Mangrove trees and mimics attracted fishes alike. We show that mangrove trees on backreefs provide habitat for shoaling and adult reef fishes in addition to serving as nursery areas, an ecosystem service otherwise lacking on backreef areas with low structural complexity.

## Introduction

Mangrove forests and coral reefs are highly productive ecosystems that provide essential socio-economic and ecological goods and services^1,2^. Typically, mangroves border tropical and subtropical coastlines and grow on soft sediments of riverbanks, brackish lagoons, or as fringing forests along sheltered coasts. They often are imbedded in a mosaic of coastal ecosystems, including seagrass beds and fringing or barrier reefs, that are connected to one another through physical and chemical processes but also through the movement of organisms^1,3,4^. Most studies on habitat use by tropical fish have focused separately on mangrove forests, seagrass meadows, or coral reefs, rather than assessing how fishes use the seascapes formed at the intersections among these systems^4,5^. Although this ecosystem-centric approach has changed as the importance of ecosystem connectivity is increasingly recognized, most studies are limited to quantifying connections between spatially separated ecosystems within a seascape^3,6–9^. Unusual seascape configurations formed by the direct intersection between different ecosystems are not often recognized as distinct. Further, the ecological interactions (e.g., biota-habitat associations) within these intersections require further investigation^10,11^. Mangroves growing in direct contact with coral reefs, subsequently referred to as ‘reef mangroves’ (also known as ‘oceanic mangroves’^12^), are examples of such intersecting ecosystems which could fulfil unique ecological services due to their specific configuration. Though occurrences of reef mangroves have been reported around the tropical and subtropical belt from the Great Barrier Reef to the Caribbean and Red Sea (^12–14^, supplementary material S4), the body of literature classifying their unique settings and investigating their ecological particularities is incomplete.

The role of coastal and estuarine mangroves as reef fish nursery areas or feeding grounds is well documented^3,15^. However, mangrove forests in different environmental settings (i.e. riverine, coastal fringing mangroves, or reef mangroves) can differ in their importance as fish feeding habitats^16^. Species composition and diversity of fish assemblages differ between the center and boundaries of mangrove forests and change throughout tidal cycles^7^. Further, the unique environmental setting of an ecosystem within a seascape (e.g. ‘estuarine mangrove’ vs ‘coastal mangrove’) was recently highlighted as a better indicator for the usage by juvenile fish when compared to ecosystem type (e.g. ‘mangrove forest’ without specification of the environmental setting^10,17^). The particular location of mangrove forests appears to influence both their impact on adjacent reefs and the way trees are used by reef fish^18,19^. In fact, reef mangrove habitat patches appear markedly different than estuarine mangroves in their utilization by reef fish^10^. It is therefore crucial to further characterize the environmental features of reef mangroves and the extent to which even individual reef mangrove trees are used by reef fish at different life stages in such settings.

Identifying habitats used by reef fish as shelter (e.g. from predators or strong water motion induced by wave action or tidal currents) is important from a conservation perspective because diverting destructive human activities away from these sites can protect valuable fish stocks^20,21^. Refugia provided by reef mangroves could be an important factor in the conservation and resilience of reef fish populations in the areas where such trees occur^22^. This includes not only nurseries for juvenile fish, but also sheltering space for large-bodied sub-adult, adult and shoaling reef fish. Climate change can reduce the architectural complexity of coral reefs^23^, thus reducing the availability of physical refugia for fish^24^. Large fish, which often fulfil critical functional roles, compete strongly for these rare large refugia^24^. Sheltering sites large enough to allow for large fish to aggregate are known to affect fish communities over extensive reef areas^25^. Therefore, identifying available sheltering space for shoals of large fish could be useful in spatial prioritization approaches aiming to conserve fish populations and preserve the ecological processes they are supporting such as nutrient cycling or herbivory, with cascading benefits for the health and integrity of entire coral ecosystems^25,26^. Availability of sheltering habitats can also have effects beyond reef systems, affecting presence of herbivorous and bioturbating reef fishes which contribute to shaping surrounding ecosystems^26,27^.

Reef mangroves could potentially provide such sheltering habitats on backreefs of otherwise low structural complexity and in proximity to reef crests. Extensive root systems, even of individual trees, could provide space not only as nurseries but also for sub-adult, adult and shoaling fish. However, globally, reef mangroves have been described and mapped mostly superficially. While mangrove ecosystems cover about 15% of coastlines globally^28^, a literature search identified six distinct locations in which reef mangroves are described fitting the context of this paper (supplementary material S4), though there is no indication of the total areal extent of these locations. Few detailed characterizations of the environmental features of these systems exists, and none of the studies consider the roles of individual trees or the extent to which reef fish use the root systems of these mangrove trees on consolidated reef substrate as habitats (supplementary material S4).

South of the Island of Viti-Levu in Laucala Bay (Fiji), numerous mangrove trees, estimated at 5–18 years of age (based on historical satellite imagery available via the Google Earth Pro Version 7.3), are growing on the backreef plateau of a fringing reef. The occurrence of these trees on consolidated substrates approximately 1 km further offshore from well-developed coastal fringe mangrove forests presents an ideal opportunity to study the ecological effects of individual mangrove trees on shallow backreefs, since early stages of establishment. Though it is unclear why the trees started to develop in this location only in recent decades, they seem to persist and spread on the backreef, given the large numbers of not only rooted seedlings but also small established trees developing in increasing distance to the larger established trees on the backreef.

Utilizing this opportunity, we quantified the extent to which individual mangrove trees established on backreefs are used as habitat by individual fish and/or by intra- and interspecific fish shoals. Additionally, we analyzed whether the utilization of individual mangrove trees on backreefs by reef fish is influenced by the individual trees’ root system perimeter, exposure to water motion, or the distance to the nearest reef crest, and whether this habitat usage differs between the seaward and leeward sides (aspect) of the trees. Deploying artificial mangrove tree mimics (hereafter mimics), we tested experimentally whether the effect of reef mangroves on reef fish assemblages is linked to properties other than the physical space and effect added by their root systems and shading available under their canopy. Our expectation was that the reef mangroves attract individual fish and fish shoals on the backreef due to their physical features (i.e., added structural complexity and shade as shown in^15,29^) and that numbers of fish would be higher closer to the nearest reef crest (closeness of habitats affects connectivity, fish assemblages and abundance as shown in^7,30^). We also anticipated that the number of fish found within the root systems would increase with increasing root system perimeter^15^ and reduced exposure to water motion^31^, and therefore expected fish numbers to be higher on the supposedly calmer leeward side of the trees compared to the seaward side^28^).

## Results

A total of 3025 individual fishes were counted (supplementary material S1), with *Lutjanus* spp. (n = 1264), *Abudefduf* spp. (n = 413), *Gnatholepsis cauerensis* (n = 263*) and Siganus spinus* (n = 245) recorded as the most abundant taxa. Mean total length (TL) of the various fish taxa ranged from ~ 1 cm (unidentified fry) to 16.64 cm (*Lutjanus* spp.) and most *Lutjanus* spp. and *Chrysiptera* spp. were likely mature based on their TL (supplementary material S2). Most fish shoals (see supplementary material S3) were heterotypic groups of *Lutjanus* spp. (232 shoals observed with a mean shoal size of 44 individuals).

### Reef mangroves aggregate fish on backreefs

**Figure 1:**
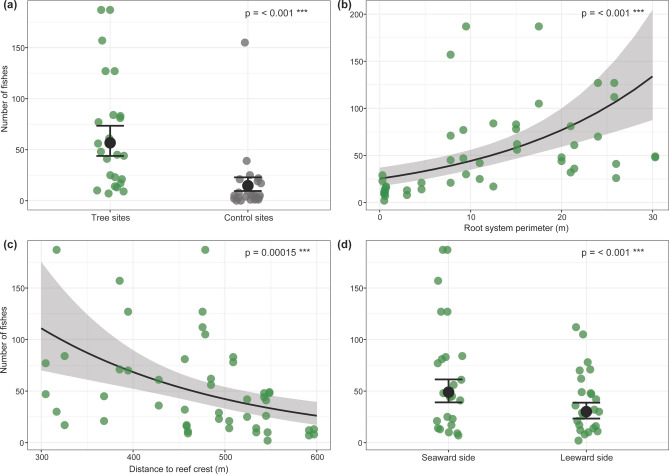
Results of the fish abundance counts (**a**) Difference in the total number of fishes observed at tree and control sites with fitted values as solid black dots (± 95% CI), and observed values as color-coded dots (tree sites: green, control sites: grey). (**b**) Relationship between the root system perimeter and the number of fish observed at mangrove trees sites (± CI) with observed values as green dots. (**c**) Relationship between the distance to the reef crest and the number of fish associated with the tree sites (± CI) with observed values as green dots. (**b**) Differences in number of fish between the leeward and seaward side of tree sites (± CI) with observed values as green dots.

The presence of individual mangrove trees on backreefs strongly influenced backreef fish abundance. Fish abundance was 3.7 times higher at back reef sites where trees were present (i.e., tree sites) compared to back reef sites without trees (i.e., control sites) (*p* < 0.001, Fig. 1a). Variance in fish abundance was also higher among tree sites than among control sites (Fig. 1a).

Fish numbers associated with natural mangrove trees were strongly and positively influenced by the outer root system perimeter, with 3.6 times more fish within the larger root systems than within smallest ones (*p* < 0.001, Fig. 1b). A threshold effect of markedly higher fish abundances was detected associated with reef system perimeters of 7.8 m or larger (Fig. 1b, coinciding with the presence of fish shoals as shown in Fig. 3d). Distance to the reef crest also influenced fish abundance (p =  < 0.001, Fig. 1c). The fitted mean value (model’s prediction of the mean response value) of number of fishes was approximately 4.4 times higher, with up to 110 individual fish found closest (300 m) to the reef crests than furthest away from it (25 fishes 600 m away from the crest, Fig. 1c). Mean fish numbers were almost double in the seaward compared to the leeward side of tree sites (*p* < 0.001, Fig. 1d). Variance in fish abundance increased with increasing root system perimeter and with decreasing distance to the reef crest and was higher at the seaward side of the trees when compared to the leeward side (Fig. 1d).

### The influence of reef mangroves on backreef fish assemblages is principally physical

**Figure 2:**
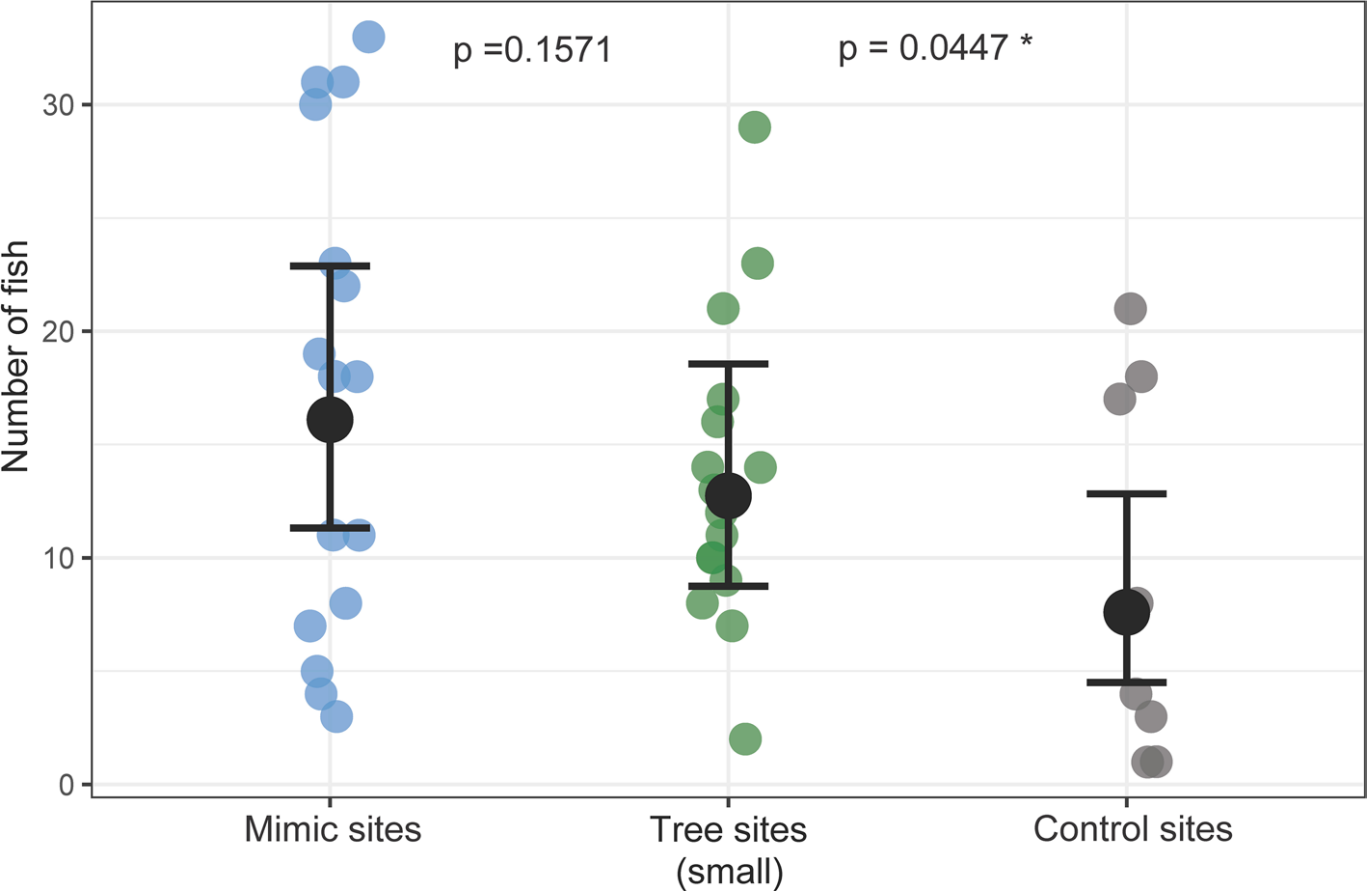
‘Number of fish’ plotted against treatments ‘Tree’ (size category small), ‘Mimic’, and ‘Control’. Observed values shown as color coded dots (mimic sites: blue, tree sites: green, control sites: grey), solid black dots represent fitted values (± 95% CI).

To disentangle whether the influence of reef mangroves on backreef fish assemblages is primarily driven by the physical or biological properties of the trees, we surveyed root systems of small natural trees and artificial tree mimics (Fig. 4e). The effect of reef mangroves on backreef fish assemblages appeared linked mostly to the physical properties of the trees (canopy shading and structural complexity added by the roots), given that fish numbers within root systems of small trees and mimics did not differ significantly (*p* = 0.1571), while in comparison, control plots showed a significantly lower number of fish (*p* = 0.0447, Fig. 2).

### Reef mangrove trees offer habitat for individual fish and fish shoals

**Figure 3:**
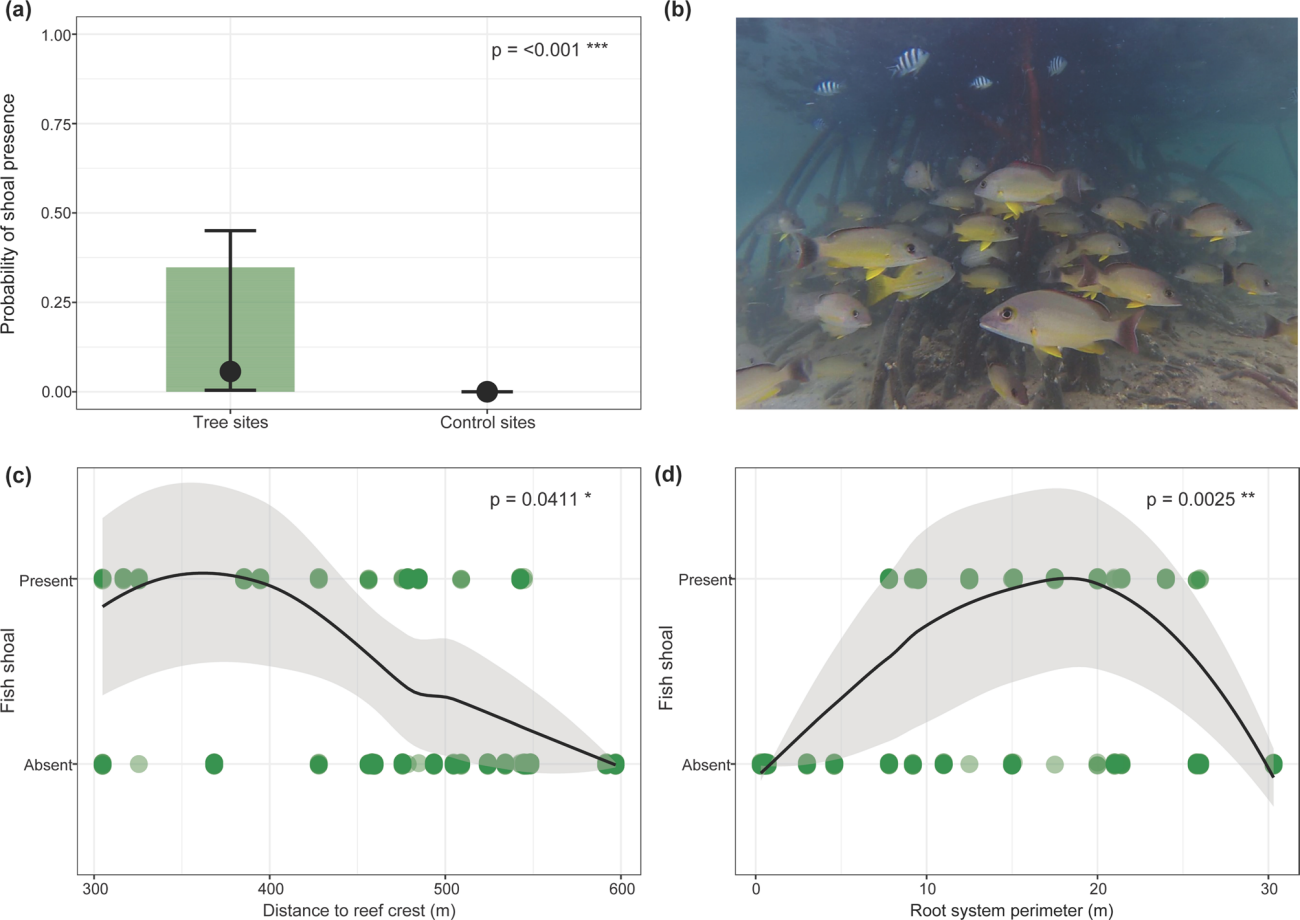
Results of quantifying fish aggregating in shoals (**a**) Difference in the probability of presence of fish shoal between tree and control sites. Observed values shown as color-coded bars (tree sites: green, control sites: no observations) whereas solid black dots and error bars represent fitted values (± 95% CI). (**b**) Shoaling Lutjanus fulvus, and a few individuals of Abudefduf sexfasciatus at a tree of medium- sized root system captured during video surveys. (**c**) Significant non-linear relationship between the presence-absence of fish shoals and the distance to the reef crest. Fitted line (± CI) and observed occurrences shown as dots (tree sites = green). (**d**) Significant non-linear relationship between the presence-absence of fish shoals and the root system perimeter. Fitted line (± CI) and observed occurrences shown as dots (tree sites = green).

The root systems of reef mangroves were regularly used as habitat and refuge by shoaling fish. Presence/absence analysis of shoaling fish from video surveys showed the probability of encountering shoaling fish on the backreef was ~ 34.8% at tree sites whereas it was zero at control sites (*p* =  < 0.001, Fig. 3a). Of all observed fish shoals (n = 298), 78% were comprised by adults of the genus *Lutjanus* (n = 232), 12% were made up of unidentified fry (with mean TL ~ 1 cm; n = 36), and 10% were comprised by conspecifics of *Abudefduf sexfasciatus* (n = 30; Fig. 3b).

The probability of presence of fish shoals was significantly and non-linearly related with both distance of a tree site to the reef crest (*p* = 0.0411, Fig. 3c) and root system perimeter (p = 0.0025, Fig. 3d). Fish shoals were present in 70% of video clips collected at the tree sites within 450 m distance to the reef crest. They were also observed in 46% of video clips collected at the tree sites between 450 and 550 m distance to the reef crest, and in none of the video clips collected at the tree sites in 550—600 m distance to the reef crest (Fig. 3c). Fish shoals were never observed within root systems < 7.8 m or > 26 m in perimeter but were present in the majority (52%) of the video clips within root systems ranging between 7.8 and 26 m (Fig. 3d).

## Discussion

Shallow backreefs, especially when infilled with sediment as in the study site, can offer limited structural complexity for reef fish compared to reef crests, forereefs, reef lagoons or mangrove lagoons^32,33^. We document a seascape configured by individual mangrove trees growing relatively sparsely over a shallow backreef and the influence of these trees on reef fish communities. Although the occurrence of mangrove trees on backreefs is not rare, their influence on the reef environment or biota has been scarcely investigated^12,14,34^. As submerged root systems of coastal mangrove forests comprise one of the most important nursery habitats for reef fish^3,29,35^, single trees may add sufficient structural complexity to congregate fish on otherwise unpopulated backreefs. Here we demonstrate that reef mangrove trees congregate both individual and shoaling fish, much like large sheltering spaces and overhangs do in deeper and more complex (e.g. forereef) habitats^36,37^. Most fish using these trees must migrate routinely to and from the forereef at least twice a day, following the tidal cycle as the backreef is fully emerged during low tide. Both the number of individual fish present at a site as well as the probability of shoaling fish occurring at a site were highest closest to the reef crest, and numbers of fish were also higher on the seaward side compared to the leeward side of trees. This supports previous conclusions regarding the effect of distance between habitats on their utilization by reef fish^18^. As expected, the number of fish using mangrove roots was directly proportional to the root system perimeter. Further, fish were attracted to the roots of small trees regardless of whether these were natural or artificially mimicked, indicating that fish are mostly influenced by the physical (rather than biochemical) properties of the trees. Single mangrove trees can thus supply familiar services (i.e. providing sheltering or nursery habitats for fish) in peculiar places (e.g. backreefs somewhat distant from the shore), contributing regulating or supporting ecosystem services^1^. These services are similar to those supplied by other types of mangrove forests, which could have important implications for effective management and conservation efforts aimed at protecting reef mangroves and/or inshore fishery resources.

Our study demonstrates that mangrove trees established on backreefs attract 3.7 times more fish than “bare” sections of the backreef (control sites). Both the number of fish and the probability of fish shoals utilizing mangrove roots as a habitat decreased with increasing distance from the reef crest. This supports the hypothesis that the distance between the ecosystem providing a habitat service for reef fish and the closest reef will influence the magnitude of the habitat service^18^, even at comparatively small scales and distances as investigated in this study. The negative relationship between a tree’s distance to the reef crest and the number of fish inhabiting its roots could furthermore be explained by the fact that the backreef flat is fully emerged during low tide. As a result, most fish left the backreef area during low tide, with only juvenile fish and snowflake morays (*Echidna nebulosi*) retreating into small, inundated crevices (pers. obs.). Hence, closer proximity to the reef crest could be beneficial at the onset of low tide in order to quickly move to other refugia available on the reef crest.

The results of the experiment suggest that the physical properties (root structure and shade provided by the canopy—as imitated by the mimics) of trees are the main properties driving the habitat provision service, rather than biochemical properties or processes, such as quality of mangrove leaves and/or detritus or differences in nutrient cycling. This is further supported by the positive relationship between fish abundance and root system perimeter. Reasons why the structure added by the mangrove trees affects fish abundance could include i) the availability of space for sheltering from predators within the root system, ii) the protection offered by the canopy shading, since solar irradiance can be an important driver of shelter use^38^, and iii) the reduced water motion. Water motion in backreef habitats is relatively low due to the wavebreak effect of the adjacent reef crest^39^. Hence, the presence of trees is arguably unlikely to substantially reduce water motion further in these habitats. However, this backreef lagoon is subject to tidal currents^40^. We had thus hypothesized that the structure provided by the root systems of the trees would further attenuate water motion and thus provide even calmer sheltering spaces for fishes. We further postulated that the side of trees opposite the reef crest (leeward) would have a lower Water Motion Index (WMI) compared to the side of the tree facing the reef crest (seaward). Accordingly, we expected the leeward side to favor larger fish numbers compared to the seaward side. Contrary to this expectation, no significant difference in water motion between the different side of trees was found by Fett et al. (*unpub. data*), indicating that the presence of the trees does not generate fixed leeward or seaward conditions where we expected. However, we observed about twice as many fish and more frequent fish aggregations on the side of the root systems directly facing the reef crest. Taken together, this means that fish sheltering in and around the root systems of the mangrove trees generally do not seek reduced water movement, but rather shade and protection from predators. Additionally, the position of fish aggregations with respect to the trees likely shortens the path for diel tidal migrations between the reef and the tree (see above) or provides more direct access to possible food sources on the reef.

Our results underline that mangrove tree roots provide habitat for fish of different species and cohorts (juveniles, subadults, or adults) during high tide. Notably, 63% of observed individuals of the genus *Lutjanus*, the genus with the highest abundance (making up more than a third of observed fish), were over 20 cm in TL (supplementary material S2). Fish in this size category correspond to either subadult or adult individuals given that *Lutjanus* species observed in the study area are known to mature between 20 and 30 cm TL^41^. Thus far, reviews of habitat use focusing on the overarching term ‘mangrove’ had found that these trees are used mainly by juvenile individuals of the genus *Lutjanus*^9^. However, studies that consider the environmental contexts found that late juveniles of *Lutjanus fulviflamma* almost exclusively utilize reef mangrove habitats as opposed to other late juveniles^10^. The studied individual mangrove trees also served as a fish nursery, with a large proportion of the observed fishes being juveniles (e.g. *Halichoeres spp*.; *Abudefduf spp.*) or fish fry (supplementary material S2). Thus, the reef mangroves studied here provide additional habitat for juvenile fish found in backreef habitats. Interestingly, compared to the roots of mangroves occurring in other geomorphic settings (e.g., fringing shorelines), which are inhabited predominantly by juvenile fish^3^, reef mangroves here appeared to also provide additional habitat for large numbers of subadult and adult fish inhabiting backreefs.

Fish shoals were commonly recorded among the mangrove root systems of trees with a perimeter of at least ~ 7 m. Fish shoals never took shelter in trees with a root system perimeter below this threshold and were never observed at control sites. Above the ~ 7 m threshold, the probability of fish shoals sheltering within root systems increased with the root system perimeter. However, among trees with root systems > 26 m in perimeter, this trend reversed. It is possible that fish shoals were present within the largest root systems but were not captured by the video cameras because of poor light and/or large distance. Shoals of *Lutjanus* spp. (n > 20 individuals) were the most frequently recorded congregating within mangrove roots. *Lutjanus* spp. are known to utilize mangrove areas as juveniles but return to the reef as adults where they often form groups or heterotypic shoals^29,42^. Space for adult Lutjanids (~ 20–40 cm TL) to shelter in shoals is limited by the availability of large overhangs, caves, or boulders^24,43^. Our study shows that single mangrove trees can fulfil this role in an otherwise fairly flat habitat. The sheltering opportunities mangroves provide may be frequented by Lutjanid shoals seeking protection from predators on the otherwise scarcely vegetated reef flats, which are known to be frequented by larger predators such as sharks^44,45^. We thus argue that the mangrove trees on backreefs attract fish not only as nursery areas but are also attractive as refuges for shoaling adult fish. The mangrove trees provide an ecosystem service that is otherwise lacking in backreef habitats with little structural complexity and increasingly scarcer in flattening reef crests and forereefs^46^.

Reef flats in general, including shallow backreef areas, are easily accessible to people and thus serve as key fishing grounds in many coastal areas worldwide^45^. On the studied backreef, fishing via hand gleaning and spearfishing occurred frequently over the duration of the study. Fish inhabiting this backreef are thus likely used as valuable sources of nutrition or income. Especially in light of ongoing global degradation of reef ecosystems and declining reef fish populations^47^, additional fish habitats in close proximity to reefs can play a role supporting juvenile and adult reef fish populations which could potentially and eventually supply fish larvae for adjacent ecosystems^19,25^.

Likewise, mangroves are subject to a variety of threats including erosion, extreme weather events such as storms, or through direct anthropogenic actions like deforestation for agricultural purposes or urban development^48^. The presence and successful establishment of *Rhizophora stylosa* on the consolidated substrates of a backreef area show this species is able to utilize a small window of opportunity between the semidiurnal tidal cycles to settle. Studies have shown that it is more difficult for mangrove seedlings to settle in areas with high water stands and in areas with consolidated sediments^49^. On the studied backreef, seedlings develop rooting strong enough to withstand the tidal currents, despite being fully submerged during high tide. Anchoring of roots on the consolidated substrates can be sturdier than anchoring in soft sediments^50^, enhancing the likelihood for the seedlings to further develop and grow in this tidal regime and to withstand other threats such as storms.

Highlighting that individual mangrove trees can attract significant numbers of fish underscores the importance of considering even small patches of mangrove stands in ecosystem management and urban planning. Though urban estuarine mangrove habitats can be important habitat for fish species^51,52^, constraints for urban rehabilitation of mangrove areas include space considerations^53^. While other factors such as turbidity and exposure to frequent disturbances in harbor areas may influence overall fish presence in fragmented patches of mangrove habitat^54^, small mangrove stands or even individual trees should be considered as potentially important shelters in areas that are found to be frequented by reef fish, in both natural and urban settings.

Fish counts obtained here through stationary visual censuses correspond to the surveyor’s field of view rather than to a pre-defined fixed area. Arguably, at large trees (i.e. with root system perimeters > 17.5 m), part of the field of vision might be obscured due to the density and complexity of the root system itself. Mangrove root complexity can be an important indicator for attractiveness of the root system to fish^55^, however, no metric of the density or complexity of the root systems was quantified in this study, which prevents us from testing whether this attribute correlates with the root system perimeter. Hence, it is possible that fish abundance within medium and large tree sites was underestimated. A human surveyor, as present in stationary point counts, could alter the behavior of fish, which retreat further into the roots as a response, leading to an underestimation of abundance. To minimize these effects, fish were given time to acclimate in the surveyor’s presence before counts were started. Reliability of the visual point count data despite presence of the surveyor can be supported by data from the video analysis. Abundance results from visual point counts register a distinct and steep increase in fish numbers at trees with a root system perimeter of 7.8 m, aligning with the same threshold registered for shoaling fish presence, which were recorded only in trees with root system perimeters of 7.8 m or more. Visual point counts conducted over several minutes also introduce the risk of double-counting individual fish. While the comparability of fish numbers between sites is likely still given, the abundance data might not reflect accurate absolute density values. Further, while using video cameras for fish surveys removes observer bias and avoids the influence of surveyor presence in wary species, the cameras used here were highly light sensitive. Thus, shading within the roots of the trees could have led to an underestimation of fish abundance, especially in the larger trees with larger root perimeters and canopy. Here too, a metric assessing root system complexity would have allowed us to potentially account for this bias. However, as most of these limitations are in turn likely to have led to an underestimation of fish abundance at the tree sites, we argue that the significances of the results presented in this study still hold.

Finally, our study only analyzed the total number of individual fish or fish shoals and did not distinguish between different species. Future studies incorporating this distinction would be needed to capture potential interspecific differences in habitat use on backreefs caused by individual mangrove trees.

## Conclusions

Individual mangrove trees attract important numbers of fish within their root systems on shallow otherwise flat backreefs. In this backreef system, mangrove trees not only act as nurseries for juvenile fish, but also as sheltering habitat for adult reef fish. This close ecological connection between individual mangrove trees and coral reef fish could be an important aspect to consider for species replenishment, especially in areas affected by overfishing or reef habitat degradation. Our results highlight the need to consider environmental context in studies addressing tropical coastal ecosystems. Further studies, including comparisons between the mangroves on these peculiar backreefs with fringing mangroves or other surrounding habitats, would help assessing the value of the respective systems. In addition, comparison with similar settings (individual mangrove trees on a backreef area) in other regions would be required to verify the generality of our results.

## Methods

### Study site

**Figure 4:**
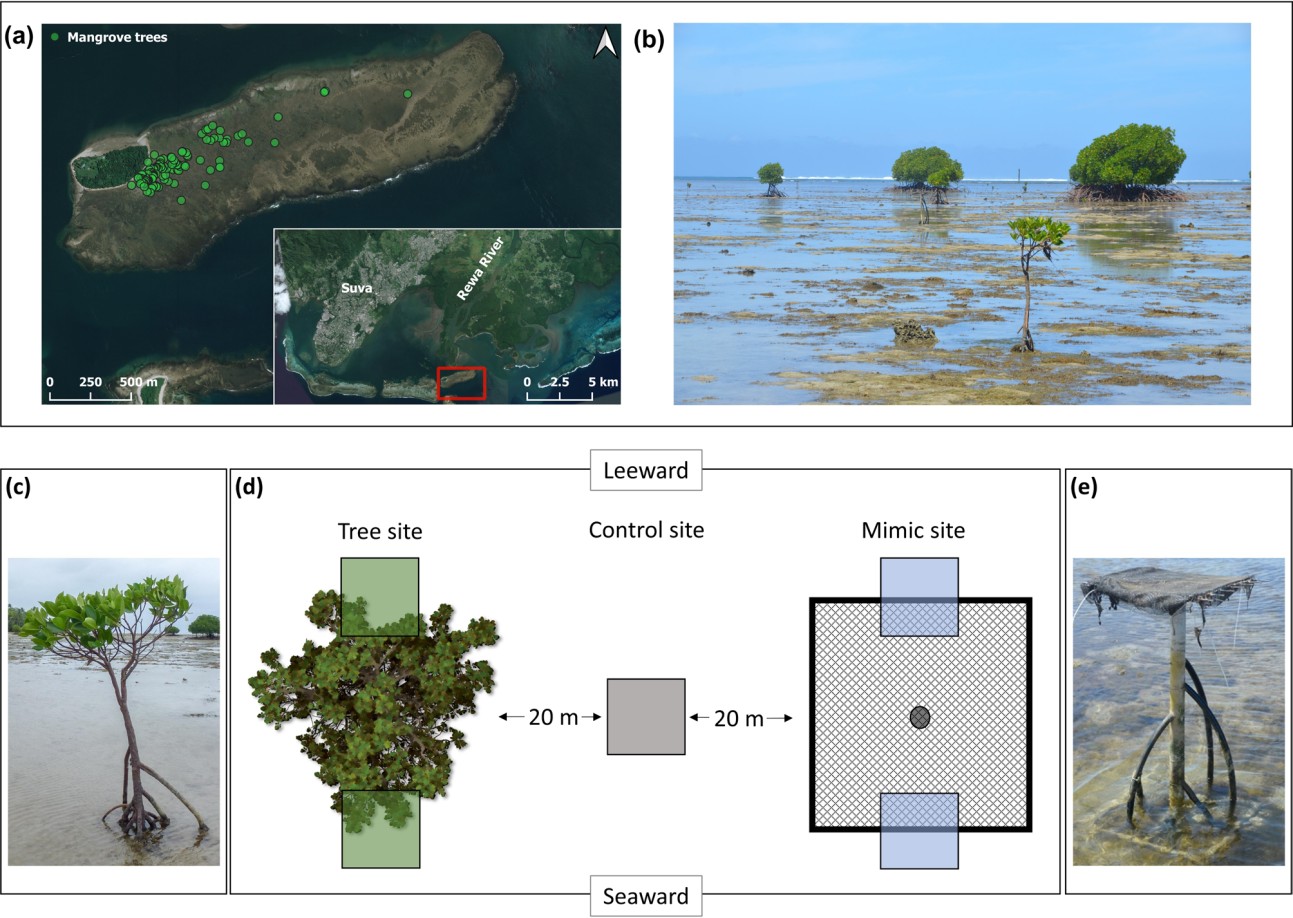
Upper row: Overview of research area (**a**) Satellite image of the research area showing the location of mangrove trees on the backreef area relative to the delta of the Rewa River and the capital city Suva. (Basemap ©2023 Microsoft, created with QGIS Development Team 2019). (**b**) Environmental setting of the backreef flat exposed during low tide, a mix of coral rock, rubble, and sand. Lower row: Experimental and sampling setup (**c**) Mangrove tree of size category ‘small’ (**d**) Schematic representation of each of the 8 sampling locations with small trees surveyed in this study. Each of these locations comprised one tree site, one control site and one mimic site. The remaining sampling locations with medium (n = 8) and large trees (n = 8) comprised only one tree site and one control site each, otherwise following the same layout scheme. Color-coded squares (green = tree site, grey = control site, blue = mimic site) depict the areas where surveys took place (i.e. leeward and seaward of the base of the trees for tree and mimic sites, and at centrally located control sides). Each sampling site within the location was located > 20 m away from every other site. (**e**) Tree mimic.

The study site is located in Laucala Bay, southeast off the island of Viti Levu (Fiji) in the South Pacific Ocean. The backreef lies ~ 8 km east of Fiji’s capital city, Suva, and within the drainage area of the Rewa River (S 18°10′23.403″, E178°31′8.722″, WGS 84). Laucala Bay and its mangrove forest are subjected to the Rewa River discharge as major source of sediments^40^. High levels of organic matter, but also heavy metals and microplastic pollution near the Suva harbor have been recorded^56,57^ and could affect all mangroves in Laucala Bay. Mangroves and the adjacent reef areas in this region are of high value to the local population for both coastal protection and food provisioning. They play a key role as habitats for fishes, and act as nutrient traps buffering the effects of the pollutants in the lagoon^58^. The western part of the backreef is elevated above sea level, forming a small island (Nukulau), and the mangrove trees studied here (*Rhizophora stylosa*) populate the eastern side of the backreef adjacent to Nukulau Island. These trees are approximately ~ 1 km apart from established mangrove forests of the Rewa River (Fig. 4a).

The substrate of the backreef comprises a heterogeneous mix of coral rock, rubble and sand, on which small patches of seagrass and macroalgae occur (e.g. *Thalassia* sp. and *Halophila* sp. mixed with the leathery macroalgae *Sargassum* sp.; Fig. 4b). The area is subjected to semidiurnal tidal cycles and large tidal ranges. It is exposed at low tide and reaches water depths of approximately 1.1 m during spring tide. During low tide, water only remains in small puddles on the backreef as well as in narrow cracks and crevices in the coral rock.

## Survey and experimental setup

Fieldwork took place from November 2018 to February 2019 covering the majority of the wet season. An initial characterization of the area accounted for 122 trees over an area of 1.8 km^2^, from which a total of 24 trees (hereafter referred to as tree sites) were selected for this study. To be considered for inclusion, each mangrove tree had to be located at least 20 m away from any other tree and at a distance of at least 100 m from Nukulau Island. The outer root system perimeter of each tree that fit these criteria was measured at the substrate level and recorded as possible variable affecting utilization by reef fish. To ensure inclusion of trees spanning the entire size range, the trees were clustered into three size classes by sorting them based on their outer root system perimeter and dividing them into three groups containing equal numbers of trees. The trees were numbered, and eight trees of each size class were then randomly chosen from this pool, resulting in a selection of small (0.3–4.6 m) medium (7.8–15.1 m) and large trees (17.5–30.3 m). Control sites (backreef sites without trees) were established 20 m perpendicular to each of the 24 chosen tree sites in parallel direction to the reef crest to ensure similar water motion levels. A pilot experiment placing cameras at different distances to the trees on the backreef had shown that even at 4 m distance to the root systems, fish were recorded only sporadically and at a frequency similar to a distance of 20 m to the root systems. Leaving 20 m between tree and control sites left us with a reasonable pool of suitable study sites distributed across the backreef to draw from.

We expected that reef mangroves would influence fish communities through both the biological and physical characteristics of the trees. We thus conducted an experiment to disentangle the physical effects of the space and effect added by the root systems of the trees and the shading available under their canopy from all biological properties (e.g. sediment microbes, biochemical compounds released from roots, bark, or fallen leaves). To this aim, we installed eight mimics constructed of welded metal tubing, plastic pipes (PE and PVC), and mosquito netting, similar to designs used in other reef fish studies^55,59^ (Fig. 4e). Mimics were crafted to imitate the average shape and structure of small trees (Fig. 4 c & e) in terms of number of first-, second-, and third-order roots (i.e. 6, 2, and 1, respectively), and the average cumulative outer root diameter (i.e. 2 m). The degree of shading provided by the canopy of small trees was measured using HOBO Pendant® Temperature/Light Data Loggers fixed to the base of the trees within the root system and the netting on the mimics was layered to provide the same degree of shading. Mimics were placed 20 m apart from control sites on the opposite side of the natural mangrove tree, so that each small tree shared a control site with their mimic counterpart, in parallel direction to the reef crest (Fig. 4d). Mimics were fixed to the substratum using 1 m metal rods and allowed to precondition (potential settling of microorganisms on the material) for two weeks before commencing any surveys.

In total, 24 sampling locations (one location being comprised of one tree site, one control site and one mimic for the small trees) were surveyed.

To investigate whether fish presence was affected by water motion (supposedly attenuated by the root system), water motion data (Water Motion Index -WMI- estimated using clod cards^60^ installed in proximity of the trees, mimics and control sites) were derived from a parallel study that aimed at evaluating the effect of the trees on their physico-chemical environment (detailed methods in Fett et al., *in prep.*).

GPS coordinates of each tree, mimic, and control site were taken with a handheld GPS device. Using a satellite basemap (©2019 Microsoft Bing Satellite Basemap) and distance measuring tools in a Geographic Information System software (QGIS^61^), the distance of each tree to the nearest point on the reef crest was determined.

## Quantifying the effect of reef mangrove trees on backreef fish assemblages

To quantify the effect of individual mangrove trees on fish abundance, stationary visual censuses were conducted at tree, control, and mimic sites. Stationary point counts deliver accurate estimates of fish density and size when targeting large aggregations like those found within mangrove roots^62^. Counts were always conducted by the same surveyor, remaining stationary one meter away from the perimeter of the root system facing towards the tree or mimic. At control sites, the surveyor remained stationary one meter away from a short (~ 10 cm) central marker pole inserted into the sediment. The marker ensured that the surveyor could recognize the exact location of the control site but was inconspicuous enough to not attract fish. Rather than counting fishes within a fixed delimited area, the surveyor counted all fish within the field of vision centered straight towards the root system or marker pole ahead. Given that all censuses were conducted within 2 h before or after high tide (~ 0.80 m), the field of vision encompassed a cone-shaped volume of ca 0.17 m^3^ and a ca 0.5 m^2^ area at the outer root line or marker pole. The surveyor waited in their stationary position for a duration of 2 min before starting the count to reduce effects on fish behavior in response to human presence. Though only abundance data was used for analysis, all fish sighted within 10 min were counted, identified to the lowest taxonomic level possible, and their total length (TL) was estimated to the nearest centimeter (summarized information available in supplementary material S1 and S2).

We used unmanned GoPro video cameras to quantify the presence of fish aggregating in shoals (i.e., groups of ≥ 20 individuals) within the root systems. Cameras were mounted onto metal rods one meter away from the root system perimeter at the leeward and seaward sides of all the tree and mimic sites as well as at the control sites. Cameras were set to record for 20 min per site. For every minute of each recording, 30-s clips were analyzed for presence or absence of fish shoals (n = 20 clips per tree site), identifying fishes to the lowest taxonomic level possible. If any of the clips contained groups of ≥ 20 individuals of the same species or heterotypic groups of ≥ 20 individuals of *Lutjanus* spp., shoaling aggregations would be considered present at the given site. 30-s clips were long enough for the surveyor to get a confident estimate on the fish abundance counts but as short as possible to reduce effort.

The combination of visual point counts and video surveys was chosen to best fit the two main aspects of the study: individual counts and shoaling behavior. Usage of video cameras is advantageous when trying to capture behavioral aspects such as shoaling, but identifying individual fish can be challenging on video due to variable light conditions, the restricted field of view and need of high water clarity^63^, which was not always given in the study area.

## Statistical analyses

To address the different research objectives established in this study, we divided our dataset into three subsets and fitted four separate Generalized Linear Mixed-Effects models (GLMMs) and one Generalized Additive Mixed-effects model (GAMM). The first subset contained all data from tree sites and control sites. The second subset contained only data from tree sites, and the third subset contained all data from ‘small’ tree sites and mimic sites. Collinearity between all predictors was assessed using a pairwise scatterplot matrix with correlation coefficient (‘pairs’ function^64^) and only non-collinear predictors (Pearson correlation coefficient [r] within the ranges of − 0.3 to 0.3) were included within the models. WMI was correlated with the variables ‘root system perimeter’ (r = − 0.4) and ‘distance to the reef crest’ (r = − 0.4) and therefore excluded from the models. *Location* was included as a random effect in all models to account for the spatial dependence among sites (tree site, control site, and mimic site) within a sampling location (Fig. 1d).

In order to determine whether fish abundance is higher at tree sites than at control sites, a negative binomial GLMM^65^ was fitted to the first data subset, modelling fish abundance as a function of *treatment* (i.e., factor with levels *tree site* and *control site*) and *distance to the reef crest* (m). To test for an effect of tree size on fish abundance, we fitted a second negative binomial GLMM to the second data subset, modelling fish abundance as a function of *root system perimeter*, *aspect* (i.e., factor with levels *leeward* and *seaward*), and *distance to the reef crest*. To disentangle the influence of the physical characteristics of the trees (i.e., added shading and structural complexity) from the effect of their biochemical characteristics on fish abundance, a third negative binomial GLMM was fitted to the third data subset, modelling fish abundance as a function of *treatment* (i.e. factor with levels *small tree site* and *mimic*), *aspect* (*leeward* vs. *seaward*) and *distance to the reef crest* (m). Aspect was later excluded from the model due to insignificant effects, which allowed for inclusion of the *treatment* factor level *control* in this model, which did not have a corresponding aspect variable.

To determine whether the presence of mangrove trees favors fish aggregations, a binomial GLMM was fitted to the first data subset to model the presence/absence of fish shoals as a function of *treatment* (*tree site* and *control site*) and *distance to the reef crest*^66^. To test whether the probability of fish shoals aggregating within root systems, or the shoal size, were influenced by the root system perimeter, a GAMM was fitted to the second data subset, because model residuals of a GLMM indicated nonlinear patterns^67^. Variables included in this GAMM were *distance to the reef crest* and *root system perimeter* (the variable *aspect* was excluded from the GAMM because it did not explain a significant amount of variability). The smoothing terms and random effect structure for the GAMM were chosen based on the Akaike Information Criterion^68^. Since no fish shoals were recorded in small trees or mimics, we abstained from developing a model comparing the effects of these treatments on fish shoaling.

Models were tested for homoscedasticity and normality inspecting plots of model residuals vs. fitted values^69,70^. If assumptions of homogeneity of variance were not met, a variance structure was incorporated in the model ^69^. This was the case for the heterogeneous variance among treatments as well as with increasing distance to the reef crest, root system perimeter, and aspect (leeward/seaward). Initial models were simplified to optimal models using stepwise single- term deletions and comparing the AIC. Models were tested for overdispersion and spatial autocorrelation^69^. Post-hoc Tukey tests were conducted when a given treatment was a significant predictor^71^.

All statistical analyses were carried out in R version 3.5.0^64^. GLMMs were fitted using the R package ‘glmmTMB’^65^ or ‘blme’^66^ and GAMMs were fitted using the R package ‘mgcv’^67^. All plots were created using ggplot2^72^.

### Ethics approval

All research was conducted in accordance with the research permit issued by the Fiji Immigration Department (permit no. PSRS1976555). Permission to conduct field work was obtained from the council and chief of Laucala village, who hold the traditional proprietary rights to the research area. Collection of fish abundance data was entirely non-invasive and therefore no animal welfare concerns arose.

### Supplementary Information

Supplementary Information.

## Data Availability

The datasets generated and/or analyzed during the current study are available in the GitHub repository, https://github.com/HannahvHammerstein/mangrovebackreef.

## References

[1] Ewel, K. C., Twilley, R. R. & Ong, J. E. Different kinds of mangrove forests provide different goods and services. *Glob. Ecol. Biogeogr. Lett.* **7**, 83–94 (1998). 10.2307/2997700

[2] Moberg, F. & Folke, C. Ecological goods and services of coral reef ecosystems. *Ecol. Econ.* **29**, 215–233 (1999). 10.1016/S0921-8009(99)00009-9

[3] Jones, D. L., Walter, J. F., Brooks, E. N. & Serafy, J. E. Connectivity through ontogeny: Fish population linkages among mangrove and coral reef habitats. *Mar. Ecol. Prog. Ser.* **401**, 245–258 (2010). 10.3354/meps08404

[4] Guannel, G., Arkema, K., Ruggiero, P. & Verutes, G. The power of three: Coral reefs, seagrasses and mangroves protect coastal regions and increase their resilience. *PLoS One* **11**, e0158094 (2016). 27409584 10.1371/journal.pone.0158094 PMC4943730

[5] Chittaro, P. M., Usseglio, P. & Sale, P. F. Variation in fish density, assemblage composition and relative rates of predation among mangrove, seagrass and coral reef habitats. *Environ. Biol. Fishes* **72**, 175–187 (2005). 10.1007/s10641-004-9077-2

[6] Sambrook, K. *et al.* Broadening our horizons: seascape use by coral reef-associated fishes in Kavieng, Papua New Guinea, is common and diverse. *Coral Reefs* **39**, 1187–1197 (2020). 10.1007/s00338-020-01954-2

[7] Dubuc, A., Waltham, N. J., Baker, R., Marchand, C. & Sheaves, M. Patterns of fish utilisation in a tropical Indo-Pacific mangrove-coral seascape New Caledonia. *PLoS One* **14**, e0207168 (2019). 31002717 10.1371/journal.pone.0207168 PMC6474647

[8] Nagelkerken, I., Bothwell, J., Nemeth, R. S., Pitt, J. M. & Van der Velde, G. Interlinkage between Caribbean coral reefs and seagrass beds through feeding migrations by grunts (Haemulidae) depends on habitat accessibility. *Mar. Ecol. Prog. Ser.* **368**, 155–164 (2008). 10.3354/meps07528

[9] Sambrook, K. *et al.* Beyond the reef: The widespread use of non-reef habitats by coral reef fishes. *Fish and Fisheries* **20**, 903–920 (2019). 10.1111/faf.12383

[10] Bradley, M., Baker, R., Nagelkerken, I. & Sheaves, M. Context is more important than habitat type in determining use by juvenile fish. *Landsc Ecol* **34**, 427–442 (2019). 10.1007/s10980-019-00781-3

[11] Stewart, H. A. *et al.* Novel coexisting mangrove-coral habitats: Extensive coral communities located deep within mangrove canopies of Panama, a global classification system and predicted distributions. *PLoS One* **17**, 1–28 (2022). 10.1371/journal.pone.0269181 PMC9200167 35704568

[12] Rützler, K. & Feller, I. C. Caribbean mangrove swamps. *Sci. Am.* **274**, 94–99 (1996).8643952 10.1038/scientificamerican0396-94

[13] Stoddart, D. R. Mangroves as successional stages, inner reefs of the Northern great barrier reef. *J. Biogeogr.* **7**, 269 (1980). 10.2307/2844633

[14] Por, F. D. & Dor, I. The hard bottom mangroves of Sinai, Red Sea. *Rapport de la Commission Internationale sur la mer Méditerranée* **23**, 145–147 (1975).

[15] Verweij, M. C. *et al.* Structure, food and shade attract juvenile coral reef fish to mangrove and seagrass habitats: A field experiment. *Mar. Ecol. Prog. Ser.* **306**, 257–268 (2006). 10.3354/meps306257

[16] Lugendo, B. R., Nagelkerken, I., Kruitwagen, G., Velde, G. V. & Der & Mgaya, Y. D.,. Relative importance of mangroves as feeding habitats for fishes; a comparison between mangrove habitats with different settings. *Bull. Marine Sci.* **80**, 497–512 (2007).

[17] Bradley, M., Nagelkerken, I., Baker, R. & Sheaves, M. Context dependence: a conceptual approach for understanding the habitat relationships of coastal marine fauna. *Bioscience* **70**, 986–1004 (2020).

[18] Mumby, P. J. Connectivity of reef fish between mangroves and coral reefs: Algorithms for the design of marine reserves at seascape scales. *Biol. Conserv.* **128**, 215–222 (2006). 10.1016/j.biocon.2005.09.042

[19] Nagelkerken, I. *et al.* Highly localized replenishment of coral reef fish populations near nursery habitats. *Mar. Ecol. Prog. Ser.* **568**, 137–150 (2017). 10.3354/meps12062

[20] Turnbull, J. W. *et al.* Key drivers of effectiveness in small marine protected areas. *Biodivers. Conserv.* **27**, 2217–2242 (2018). 10.1007/s10531-018-1532-z

[21] Friedlander, A. M., Brown, E. K., Jokiel, P. L., Smith, W. R. & Rodgers, K. S. Effects of habitat, wave exposure, and marine protected area status on coral reef fish assemblages in the Hawaiian archipelago. *Coral Reefs* **22**, 291–305 (2003). 10.1007/s00338-003-0317-2

[22] Lenihan, H. S. *et al.* Cascading of habitat degradation: oyster reefs invaded by refugee fishes escaping stress. *Ecol. Appl.* **11**, 764–782 (2001). 10.1890/1051-0761(2001)011[0764:COHDOR]2.0.CO;2

[23] Bozec, Y., Alvarez-Filip, L. & Mumby, P. J. The dynamics of architectural complexity on coral reefs under climate change. *Glob. Chang. Biol.* **21**, 223–235 (2015). 25099220 10.1111/gcb.12698

[24] Kerry, J. T. & Bellwood, D. R. Competition for shelter in a high-diversity system: Structure use by large reef fishes. *Coral Reefs* **35**, 245–252 (2016). 10.1007/s00338-015-1362-3

[25] Khan, J. A., Goatley, C. H. R., Brandl, S. J., Tebbett, S. B. & Bellwood, D. R. Shelter use by large reef fishes: long-term occupancy and the impacts of disturbance. *Coral Reefs* **36**, 1123–1132 (2017). 10.1007/s00338-017-1604-7

[26] Waechter, L. S., Luiz, O. J., Leprieur, F. & Bender, M. G. Functional biogeography of marine vertebrates in Atlantic Ocean reefs. *Divers. Distrib.*10.1111/ddi.13430 (2021). 10.1111/ddi.13430

[27] Madin, E. M. P. *et al.* Multi-trophic species interactions shape seascape-scale coral reef vegetation patterns. *Front. Ecol. Evol.*10.3389/fevo.2019.00102 (2019). 10.3389/fevo.2019.00102

[28] IUCN. Red List of Mangrove Ecosystems. https://www.iucn.org/resources/conservation-tool/iucn-red-list-ecosystems/red-list-mangrove-ecosystems.

[29] Rooker, J. R. *et al.* Seascape connectivity and the influence of predation risk on the movement of fishes inhabiting a back-reef ecosystem. *Ecosphere*10.1002/ecs2.2200 (2018). 10.1002/ecs2.2200

[30] Martin, T. S. H. *et al.* Effective protection of fish on inshore coral reefs depends on the scale of mangrove-reef connectivity. *Mar. Ecol. Prog. Ser.* **527**, 157–165 (2015). 10.3354/meps11295

[31] Johansen, J. L., Bellwood, D. R. & Fulton, C. J. Coral reef fishes exploit flow refuges in high-flow habitats. *Mar. Ecol. Prog. Ser.* **360**, 219–226 (2008). 10.3354/meps07482

[32] Hopley, D., Smithers, S. G. & Parnell, K. E. *The Geomorphology of the Great Barrier Reef - Development, Diversity, and Change* (Cambridge University Press, 2007).

[33] Harborne, A. R. *et al.* The functional value of Caribbean coral reef, seagrass and mangrove habitats to ecosystem processes. *Adv. Mar. Biol.* **50**, 57–189 (2006). 16782451 10.1016/S0065-2881(05)50002-6

[34] Woodroffe, C. D. Mangroves and coral reefs: David Stoddart and the Cambridge physiographic tradition. *Atoll. Res. Bull.* **2018**, 121–145 (2018).

[35] Nagelkerken, I. *et al.* Importance of mangroves, seagrass beds and the shallow coral reef as a nursery for important coral reef fishes, using a visual census technique. *Estuar Coast Shelf Sci.* **51**, 31–44 (2000). 10.1006/ecss.2000.0617

[36] Fukunaga, A., Kosaki, R. K., Pascoe, K. H. & Burns, J. H. R. Fish assemblage structure in the Northwestern Hawaiian Islands is associated with the architectural complexity of coral-reef habitats. *Diversity (Basel)* **12**, 430 (2020). 10.3390/d12110430

[37] Harborne, A. R., Mumby, P. J. & Ferrari, R. The effectiveness of different meso-scale rugosity metrics for predicting intra-habitat variation in coral-reef fish assemblages. *Environ. Biol. Fishes* **94**, 431–442 (2012). 10.1007/s10641-011-9956-2

[38] Kerry, J. T. & Bellwood, D. R. The functional role of tabular structures for large reef fishes: avoiding predators or solar irradiance?. *Coral Reefs* **34**, 693–702 (2015). 10.1007/s00338-015-1275-1

[39] Fulton, C. J. & Bellwood, D. R. Wave-induced water motion and the functional implications for coral reef fish assemblages. *Limnol. Oceanogr.* **50**, 255–264 (2005). 10.4319/lo.2005.50.1.0255

[40] Singh, A. & Aung, T. Salinity, temperature and turbidity structure in the Suva Lagoon Fiji. *Am. J. Environ. Sci.* **4**, 266–275 (2008). 10.3844/ajessp.2008.266.275

[41] FAO. *The Living Marine Resources of the Western Central Pacific - FAO Species Identification Guide for Fishery Purposes*. (Food and Agriculture organization of the United Nations, 2001).

[42] Ehrlich, P. R. & Ehrlich, A. H. Coevolution: heterotypic schooling in Caribbean reef fishes. *Am. Nat.* **107**, 157–160 (1973). 10.1086/282823

[43] Potts, G. W. The schooling ethology of Lutianus monostigma (Pisces) in the shallow reef environment of Aldabra. *J. Zool.* **161**, 223–235 (1970). 10.1111/j.1469-7998.1970.tb02037.x

[44] Bauman, A. G. *et al.* Fear effects associated with predator presence and habitat structure interact to alter herbivory on coral reefs. *Biol Lett* **15**, 20190409 (2019). 31573428 10.1098/rsbl.2019.0409 PMC6832174

[45] Harborne, A. R. The ecology, behaviour and physiology of fishes on coral reef flats, and the potential impacts of climate change. *J. Fish Biol.* **83**, 417–447 (2013). 23991866 10.1111/jfb.12203

[46] Magel, J. M. T., Burns, J. H. R., Gates, R. D. & Baum, J. K. Effects of bleaching-associated mass coral mortality on reef structural complexity across a gradient of local disturbance. *Sci. Rep.* **9**, 1–12 (2019). 30792432 10.1038/s41598-018-37713-1 PMC6385266

[47] Jones, G. P., McCormick, M. I., Srinivasan, M. & Eagle, J. V. Coral decline threatens fish biodiversity in marine reserves. *Proc. Nat. Acad. Sci.* **101**, 8251–8253 (2004). 15150414 10.1073/pnas.0401277101 PMC419589

[48] Goldberg, L., Lagomasino, D., Thomas, N. & Fatoyinbo, T. Global declines in human-driven mangrove loss. *Glob. Chang. Biol.* **26**, 5844–5855 (2020). 32654309 10.1111/gcb.15275 PMC7540710

[49] Balke, T. *et al.* Windows of opportunity: Thresholds to mangrove seedling establishment on tidal flats. *Mar. Ecol. Prog. Ser.* **440**, 1–9 (2011). 10.3354/meps09364

[50] Boizard, S. D. & Mitchell, S. J. Resistance of red mangrove (Rhizophora mangle L.) seedlings to deflection and extraction. *Trees* **25**, 371–381 (2011). 10.1007/s00468-010-0512-z

[51] Peters, J. R., Yeager, L. A. & Layman, C. A. Comparison of fish assemblages in restored and natural mangrove habitats along an urban shoreline. *Bull. Mar. Sci.* **91**, 125–139 (2015). 10.5343/bms.2014.1063

[52] Waltham, N. J., McCann, J., Power, T., Moore, M. & Buelow, C. Patterns of fish use in urban estuaries: engineering maintenance schedules to protect broader seascape habitat. *Estuar Coast Shelf Sci.* **238**, 106729 (2020). 10.1016/j.ecss.2020.106729

[53] Friess, D. A. Mangrove rehabilitation along urban coastlines: a Singapore case study. *Reg. Stud. Mar. Sci.* **16**, 279–289 (2017).

[54] Clynick, B. & Chapman, M. G. Assemblages of small fish in patchy mangrove forests in Sydney Harbour. *Mar. Freshw. Res.* **53**, 669–677 (2002). 10.1071/MF00147

[55] Cocheret de la Morinière, E., Nagelkerken, I., van der Meij, H. & van der Velde, G. What attracts juvenile coral reef fish to mangroves habitat complexity or shade?. *Mar. Biol.* **144**, 139–145 (2004). 10.1007/s00227-003-1167-8

[56] Ferreira, M., Thompson, J., Paris, A., Rohindra, D. & Rico, C. Presence of microplastics in water, sediments and fish species in an urban coastal environment of Fiji, a Pacific small island developing state. *Mar. Pollut. Bull.* **153**, 110991 (2020). 32275540 10.1016/j.marpolbul.2020.110991

[57] Pratap, A., Mani, F. S. & Prasad, S. Heavy metals contamination and risk assessment in sediments of Laucala Bay, Suva Fiji. *Mar. Pollut. Bull.* **156**, 111238 (2020). 32510382 10.1016/j.marpolbul.2020.111238

[58] Atkinson, S. C. *et al.* Prioritising mangrove ecosystem services results in spatially variable management priorities. *PLoS One* **11**, e0151992 (2016). 27008421 10.1371/journal.pone.0151992 PMC4805192

[59] Nagelkerken, I. & Faunce, C. H. Colonisation of artificial mangroves by reef fishes in a marine seascape. *Estuar Coast Shelf Sci.* **75**, 417–422 (2007). 10.1016/j.ecss.2007.05.030

[60] Doty, M. S. Measurement of water motion in reference to benthic algae growth. *Botanica Marina* **14**, 32–35 (1971). 10.1515/botm.1971.14.1.32

[61] QGIS Development Team. QGIS Geographic Information System. Preprint at http://qgis.osgeo.org (2021).

[62] Colvocoresses, J. & Acosta, A. A large-scale field comparison of strip transect and stationary point count methods for conducting length-based underwater visual surveys of reef fish populations. *Fish Res.* **85**, 130–141 (2007). 10.1016/j.fishres.2007.01.012

[63] Murphy, H. M. & Jenkins, G. P. Observational methods used in marine spatial monitoring of fishes and associated habitats: a review. *Marine Freshw. Res.* **61**, 236 (2010). 10.1071/MF09068

[64] R Development Core Team. R: A language and environment for statistical computing. *Vienna, Austria* Preprint at R Foundation for Statistical Computing, Vienna, Austria. ISBN 3–900051–07–0, URL http://www.R-project.org. (2020).

[65] Brooks, M. E. *et al.* glmmTMB balances speed and flexibility among packages for zero-inflated generalized linear mixed modeling. *R J* **9**, 378–400 (2017). 10.32614/RJ-2017-066

[66] Chung, Y., Rabe-Hesketh, S., Dorie, V., Gelman, A. & Liu, J. A nondegenerate penalized likelihood estimator for variance parameters in multilevel models. *Psychometrika* **78**, 685–709 (2013). 24092484 10.1007/s11336-013-9328-2

[67] Wood, S. N. *Generalized Additive Models: An Introduction with R* (Chapman and Hall, CRC, 2017).

[68] Sakamoto, Y., Ishiguro, M. & Kitagawa, G. Akaike information criterion statistics. *Dordrecht The Netherlands: D. Reidel* **81**, 26853 (1986).

[69] Zuur, A., Ieno, E. N., Walker, N., Saveliev, A. A. & Smith, G. M. *Mixed Effects Models and Extensions in Ecology with R* (Springer Science & Business Media, 2009).

[70] Peña, E. A. & Slate, E. H. Global validation of linear model assumptions. *J. Am. Stat. Assoc.* **101**, 341–354 (2006). 20157621 10.1198/016214505000000637 PMC2820257

[71] Day, R. W. & Quinn, G. P. Comparisons of treatments after an analysis of variance in ecology. *Ecol Monogr* **59**, 433–463 (1989). 10.2307/1943075

[72] Wickham, H. *Ggplot2: Elegant Graphics for Data Analysis* (Springer, 2016).

