# Supplementary material for: Individual mangrove trees provide alternative reef fish habitat on backreefs
Source: Sci Rep. 2024 Aug 10;14:18574. doi: 10.1038/s41598-024-69524-y (PMC11316738; doi:10.1038/s41598-024-69524-y)
Supplement: Supplementary file 1 — Supplementary Information. [file 41598_2024_69524_MOESM1_ESM.pdf]

Supplementary Material

Individual mangrove trees provide alternative reef fish habitat on backreefs

Hannah von Hammerstein [1,2,3]*, Theresa-Marie Fett [1,2], Sebastian CA Ferse [1,2,4], Véronique Helfer [2], Stuart Kininmonth [5,6], Sonia Bejarano [2]

[1] Faculty of Biology and Chemistry (FB2), University of Bremen, Bibliothekstraße 1, 28359 Bremen, Germany

[2] Leibniz Centre for Tropical Marine Research (ZMT), Fahrenheitstraße 6, 28359, Bremen, Germany

[3] Department of Geography & Environment, University of Hawai'i at Mānoa, Honolulu, 96822, Hawai'i, USA

[4] present address: Faculty of Fisheries and Marine Sciences, Bogor Agricultural University (IPB), Jl. Agatis 1, 16680 Bogor, Indonesia

[5] School of Marine Studies, The University of the South Pacific, Suva, Fiji

[6] Heron Island Research Station, The University of Queensland, Australia

Corresponding author:

Hannah v. Hammerstein

S1: Number of individuals per taxa (identification was possible at distinct level; from species to family level) observed in fish counts.

**Taxa**	**No. of individuals (sum)**
Lutjanus spp. *(comprised largely of L. fulviflamma, L. fulvus, and L. ehrenbergii)*	1264
Abudefduf spp.	413
Gnatholepsis cauerensis	263
Siganus spinus	245
Halichoeres spp.	218
Chrysiptera spp.	210
Lethrinus harak	61
Sphyraena spp.	50
Mulloidichthys flavolineatus	43
Valenciennea sexguttatus	17
Unidentified fry	137
Others	104
**Total**	**3025**

S2: Mean and range (min-max) of total body length (TL) for the taxa observed in fish counts. Estimated TL at maturity recovered from literature is also provided.

Taxa	Mean TL (cm)	TL range (cm)	Estimated TL at maturity
Lutjanus spp.	16.64	3-25	*L. fulviflamma*: 20 – 25 cm*; L. fulvus*: 20 – 30 cm; *L. ehrenbergii:* ~ 12cm (1)
Mulloidichthys flavolineatus	14.05	5-20	20.8 (2)
Sphyraena spp.	12.50	10-15	Sphyraena *barracuda:* 66 (2)
Lethrinus harak	11.11	5-20	19.5 (2)
Siganus spinus	9.90	3-20	-
Chrysiptera spp.	9.01	1-20	-
Halichoeres spp.	8.43	1-20	*Halichoeres hortulanus*: 12.8 (2)
Abudefduf spp.	7.33	1-20	*Abudefduf saxatilis:* 15 (2)
Gnatholepsis cauerensis	5.80	1-15	-
Unidentified fry	1.0	1	-

References

(1) FAO. The Living Marine Resources of the Western Central Pacific - FAO species identification guide for fishery purposes. Volume 5 B. Carpenter KE, Niem VH, editors. Food and Agriculture organization of the United Nations; 2001.

(2) Froese R, Pauly D. FishBase [Internet]. [cited 2023 Oct 2]. Available from: https://www.fishbase.org

S3: Taxa composition of observed shoaling fish, maximal & mean shoal sizes (min shoal size was set to 20 individuals), number of shoals per taxa, and total sum of observed shoaling individuals of each taxa.

Taxa	Max. shoal size	Mean shoal size	Number of shoals	Sum of shoaling individuals
***Lutjanus* spp.**	190	44	232	10215
**Unidentified fry**	110	62	36	2231
Abudefduf sexfasciatus	85	43	30	1303

S4: Literature overview

Throughout the twentieth century, various occurrences of mangroves in association with coral reefs (sometimes referred to as "reef mangroves" or "oceanic mangroves") have been reported and investigated (Fig. S4).

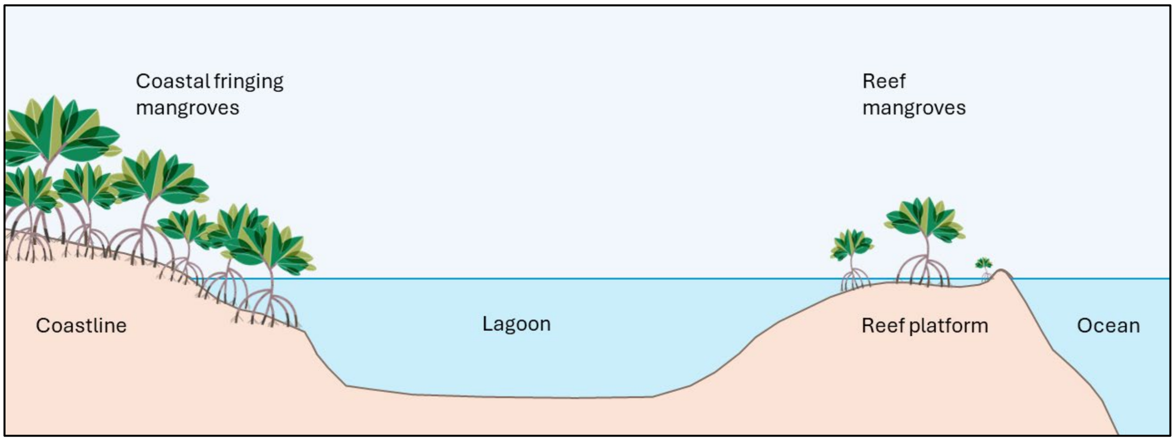

Fig. S4 Bathymetric profile of a tropical lagoon with a dense coastal mangrove forest near the shore (left) and reef mangroves situated on a reef platform offshore (right). Modified after Rützler and Feller (1996).

The geographic range of these accounts spans around the subtropical belt, from the Great Barrier Reef to the Caribbean and the Red Sea (Por & Dor, 1975; Rützler & Feller, 1996; Stoddart, 1980). These reports are mainly based on observations, repetitive mapping, and aerial photography of the areas. Researchers studied the changes in these mangrove-reef habitats over time, leading to several hypotheses about their origin and mechanisms of establishment of mangrove covered reef flats and islands (Spender, 1930; Steers & Lofthouse, 1940; Stoddart, 1978; Woodroffe, 2018). Some hypotheses imply that reefs undergo successional stages including a 'mangrove stage' in mature reefs, in which mangroves settle on the reef flat, slowing water movement and trapping sediment, ultimately leading to the formation of land (Fairbridge & Teichert, 1948; Spender, 1930; Stoddart, 1980). Others argue that the occurrences of mangroves on reef flats are opportunistic and a response to local substrate and wave energy conditions (Steers & Lofthouse, 1940; Woodroffe, 2018). Most of the studies are descriptive, though a few investigate particular ecological aspects such as biodiversity or ecological roles of mangrove species (Rogers, 2017; Stearn, 1958). Notably, the term 'Coexisting Mangrove-Coral Habitat' (CMC) has been used in recent studies to classify corals growing on mangrove roots or under mangrove canopy (Stewart et al. 2022). This constellation provides coral with a certain level of shelter from environmental stressors and has increasingly been studied in light of climate change exacerbating such stressors (Rogers and Herlan, 2012; Yates et al., 2014; Scavo Lord et al., 2020). An overview of the body of literature on mangroves and reef associations is provided in Table S4, disentangling the different mangrove/coral interaction contexts, and identifying those that could be considered 'reef mangroves' in the context of this paper. None of the studies identified in this review focus on the role of individual mangrove trees, and none of the studies on reef mangroves are investigating their role as reef fish habitat.

Table S4: Review of literature on mangroves and coral reefs in direct proximity or direct contact with each other, comparing location, aspect studied, scale at which the system was studied, and the particular mangrove/coral interaction context. Any mangrove descriptions matching 'reef mangroves' as defined in this paper are highlighted in bold/italic and marked with an asterisk in the 'Mangrove/Coral Interaction Context' column.

Author(s)	Title	Year	Location	Aspect Studied	Scale	Mangrove/Coral Interaction Context
Almy et al.	***Shallow-water stony corals of Puerto Rico***	***1963***	***Puerto Rico***	***Interactions between mangrove ecosystems and their coral associates.***	***Ecosystem-wide (Puerto Rico mangrove and reef systems)***	***Mangroves growing on reef flats*; Corals growing within mangrove root system***
Bengtsson et al.	Corals of the genus Porites are a locally abundant component of the epibiont community on mangrove prop roots at Calabash Caye, Turneffe Atoll, Belize	2019	Belize	Interactions between mangroves and corals, highlighting biodiversity implications.	Ecosystem-wide (Belize mangrove and reef systems)	Corals growing within mangrove root system
Bradley et al.	Context is More Important Than Habitat Type in Determining Use by Juvenile Fish	2019	North-eastern Australia (Great Barrier Reef)	Importance of habitat type in structuring juvenile fish assemblages.	Ecosystem-wide but split by habitat-type and environmental context	Mentions 'marine mangroves' as mangroves on rocky substrate (not further classified)
Buob	Characterization of Red Mangrove Proproot Epibiont Communities of St. Johns USVI	2019	US Virgin Islands	Recovery of epibiont communities on mangroves after Hurricane	Ecosystem-wide (US Virgin Islands mangrove and reef systems)	Corals growing within mangrove root system

Camp et al.	Mangrove and Seagrass Beds Provide Different Biogeochemical Services for Corals Threatened by Climate Change	2016	Seychelles, Sulawesi, British West Indies	Global patterns of mangrove-coral interactions with regards to biochemical services provided by mangroves	Ecosystem-wide (Seychelles mangrove and reef systems)	Corals growing within mangrove root system
Cocheret De La Morinière et al.	Post-settlement Life Cycle Migration Patterns and Habitat Preference of Coral Reef Fish	2002	Curacao, Netherlands Antilles	Nursery role of mangroves and seagrass beds for coral reef fish	Ecosystem-wide (Coral reef fish in Curacao mangroves and seagrass beds)	General interactions between mangroves and reefs in direct proximity
Fairbridge and Teichert	***The Role of Mangroves in Coastal Geomorphology***	***1948***	***North-eastern Australia (Great Barrier Reef)***	***Classification of ecosystems, contribution of mangroves to coastal land formation and sediment trapping***	***Ecosystem-wide (Various coastal geomorphological settings)***	***General interactions between mangroves and reefs in direct proximity, Mangroves growing on reef flats ****
Farnsworth and Ellison	Scale-Dependent Spatial and Temporal Variability in Biogeography of Mangrove Root Epibiont Communities	1996	Belize	Ecological roles of Caribbean mangroves in supporting coral reef health.	Ecosystem-wide (Belize mangrove and reef systems)	Corals growing within mangrove root systems
Hamylton et al.	***Mangrove expansion on the low wooded islands of the Great Barrier Reef***	***2023***	***North-eastern Australia (Great Barrier Reef)***	***Measuring forest expansion in the northern Great Barrier Reef.***	***Ecosystem-wide***	***Mangrove forests expanding onto reef flats ****

Hernández Fernández	Mangrove and coral reef dynamics in the Caribbean	2015	Cuba	Dynamics between mangroves and coral reefs.	Ecosystem-wide (Cuba mangrove and reef systems)	Corals growing within mangrove root system
Jeffrey et al.	***Spatial Patterns in Benthic Composition of Nearshore Seascapes and Implications for Scarid Populations and Fisheries in La Parguera, SW Puerto Rico***	***2010***	***Puerto Rico***	***Characterization of benthic composition***	***Ecosystem-wide (Puerto Rico mangrove and reef systems)***	***Mangroves growing on reef flats****
Kathiresan & Bingham	Biology of Mangroves and Mangrove Ecosystems	2001	Global	Biological and ecological aspects of mangroves	Ecosystem-wide (Global mangrove ecosystems)	General interactions between mangroves and reefs in direct proximity
Kellogg et al.	Identifying mangrove-coral habitats in the Florida Keys	2020	Florida Keys	Characterizing coral-mangrove habitats and their role in protecting corals from environmental stressors	Ecosystem-wide (Florida Keys mangrove and reef systems)	Corals growing within mangrove root systems or under mangrove canopy
Loya	The Red Sea coral Stylophora pistillata is an r strategist	1976	Northern Red Sea	Health of coral reefs associated with mangrove ecosystems.	Ecosystem-wide (Northern Red Sea mangrove and reef systems)	Corals growing within mangrove root system
Macintyre et al.	A general biological and geological survey of the rims of ponds in the major mangrove islands of the Pelican Cays, Belize	2000	Cuba	Reef sedimentation and contributions of mangroves.	Ecosystem-wide (Cuba mangrove and reef systems)	General interactions between mangroves and reefs in direct proximity

Maggioni et al.	The Bouraké semi-enclosed lagoon (New Caledonia)– a natural laboratory to study the lifelong adaptation of a coral reef ecosystem to extreme environmental conditions	2021	Sulawesi	Characterization of ecosystem with mangroves and coral in direct proximity under extreme conditions	Ecosystem-wide (Sulawesi mangrove and reef systems)	General interactions between mangroves and reefs in direct proximity
Por and Dor	***The hard bottom mangroves of Sinai, Red Sea***	***1975***	***Red Sea, Indian Ocean***	***Distribution and ecological roles of mangrove swamps***	***Ecosystem-wide (Red Sea and Indian Ocean mangrove swamps)***	***Mangroves growing on rocky coral bottoms ****
Rogers	High Diversity and Abundance of Scleractinian Corals Growing on and Near Mangrove Prop Roots	2009	US Virgin Islands	Diversity and abundance of corals on mangrove prop roots	Individual organisms (Corals on mangrove prop roots in US Virgin Islands)	Corals growing within mangrove root systems
Rogers	A Unique Coral Community in the Mangroves of Hurricane Hole, St. John, US Virgin Islands	2017	US Virgin Islands	Coral biodiversity in mangroves and their role as refuges from bleaching	Ecosystem-wide (US Virgin Islands mangroves)	Corals growing within mangrove root systems
Rogers and Herlan	Life on the edge: corals in mangroves and climate change	2012	US Virgin Islands	Ecological interactions between mangroves and corals.	Ecosystem-wide (US Virgin Islands mangrove and reef systems)	Corals growing within mangrove root system

Rützler and Feller	***Carribean Mangrove Swamps***	***1996***	***Belize***	***Unique adaptations and ecological interactions of mangroves in Twin Cays.***	***Ecosystem-wide (Twin Cays mangrove ecosystem)***	***Mangroves growing on reef flats ****
Scavo Lord et al.	Multi-Year Viability of a Reef Coral Population Living on Mangrove Roots Suggests an Important Role for Mangroves in the Broader Habitat Mosaic of Corals	2020	Belize	Protective role of mangroves in coral reef ecosystems.	Ecosystem-wide (Belize mangrove and reef systems)	Corals growing within mangrove root system
Spender	***Island-Reefs of the Queensland Coast***	***1930***	***North-eastern Australia (Great Barrier Reef)***	***Geomorphological development of coral reefs, including mangrove stages***	***Ecosystem-wide (Various coral reefs)***	***Development of reefs with mangrove stages ****
Stearn	A Key to West Indian Mangroves	1958	West Indies	Identification and ecological roles of mangrove species	Ecosystem-wide (West Indian mangrove species)	General interactions between mangroves and reefs in direct proximity
Steers and Lofthouse	***The Nature and Origin of Coral Reef Islands***	***1977***	***Various***	***Origins and development stages of coral reefs, including the role of mangroves.***	***Ecosystem-wide (Various coral reefs)***	***Development of reefs with mangrove stages ****

Stewart et al.	Novel Coexisting Mangrove-Coral Habitats	2022	Panama	Description and categorization of mangrove-coral habitats	Ecosystem-wide (Mangrove-coral habitats in Panama)	Corals growing within mangrove root systems or under mangrove canopy
Stoddart	Coral Reefs: Research Methods	1980	Caribbean, Northern Australia (Great Barrier Reef)	Review of research methods for studying coral reefs including mangrove interactions	Ecosystem-wide (Caribbean and Great Barrier Reef coral reefs)	General interactions between mangroves and reefs in direct proximity
Stoddart	***Mangroves as Successional Stages, Inner Reefs of the Northern Great Barrier Reef***	***1980***	***North-eastern Australia (Great Barrier Reef)***	***Dynamic relationships between coral reefs and mangrove ecosystems***	***Ecosystem-wide (Caribbean coral reefs and mangroves)***	***Development of reefs with mangrove stages ****
Woodroffe	Mangrove response to sea level rise: palaeoecological insights from macrotidal systems in northern Australia	2018	Northern Australia (Great Barrier Reef)	Sedimentation processes and geomorphology of mangroves in various settings	Ecosystem-wide (Global mangrove ecosystems)	General interactions between mangroves and reefs in direct proximity
Yamano et al.	***Holocene Sea-Level Change and Evolution of a Mixed Coral Reef and Mangrove System at Iriomote Island, southwest Japan***	***2019***	***Japan***	***Chronological development of mixed reef-mangrove systems under sea-level changes***	***Ecosystem-wide (Mixed coral reef and mangrove systems in Japan)***	***Mangroves growing on fringing reefs*, mangroves growing on backreefs ****

Yates et al.	Mangrove habitats provide refuge from climate change for reef-building corals	2014	US Virgin Islands	Characterization of mangroves as a coral refuge from climatic stressors	Ecosystem-wide (US Virgin Islands mangrove and reef systems)	Corals growing within mangrove root systems or under mangrove canopy
